# Causal relationship between obesity and anorectal abscess: a Mendelian randomization study

**DOI:** 10.3389/fmed.2024.1437849

**Published:** 2024-06-21

**Authors:** XiaoYu Zeng, HanYu Wang, Yang Deng, ZhiYu Deng, Wei Bi, Hao Fu

**Affiliations:** ^1^Clinical Medical College, Chengdu University of Traditional Chinese Medicine, Chengdu, China; ^2^Hospital of Chengdu University of Traditional Chinese Medicine, Chengdu, China

**Keywords:** Mendelian randomization, obesity, anorectal abscess, causal association, GWAS

## Abstract

**Background:**

Observational studies have indicated that obesity is a risk factor for anorectal abscess (ARB). However, it remains unclear whether a causal genetic relationship exists between obesity and ARB.

**Methods:**

Univariate and multivariate Mendelian randomization (MR) were conducted using data from a large, published genome-wide association study (GWAS) of European ancestry to infer a causal relationship between obesity and ARB. Inverse variance weighted (IVW) analysis served as the primary analysis method, with results reported as odds ratios (OR).

**Results:**

MR analysis revealed that body mass index (BMI) positively affects ARB (OR 1.974, 95% confidence interval (CI) 1.548–2.519, *p* = 4.34 × 10^−8^). The weighted median method (OR = 1.879, 95% CI 1.248–2.829, *p* = 0.002) and Bayesian model averaging (BMA) (OR = 1.88, 95% CI 1.477–2.392, *p* = 2.85 × 10^−7^) also demonstrated consistent results. Subsequently, the impact of several obesity-related characteristics on ARB was assessed. Body fat percentage (BF), whole body fat mass (FM), waist circumference (WC), and hip circumference (HC) were found to be causally associated with an increased risk of ARB. However, these associations vanished after adjusting for BMI effects.

**Conclusion:**

The study confirms a positive causal effect of obesity on ARB, highlighting that reasonable weight control is an important strategy to reduce the incidence of ARB.

## Introduction

Obesity, often characterized by excess body weight and excessive growth of adipose tissue, is typically evaluated by body mass index (BMI) in clinical practice (16, 17). Over the past 30 years, the prevalence of obesity has steadily increased worldwide, becoming a significant health issue for adults, children, and adolescents (27). By 2025, the global prevalence of obesity is projected to reach 18% for men and 21% for women (26). In addition, obesity is a recognized risk factor for the development of comorbid conditions such as cardiovascular disease, type 2 diabetes mellitus, malignancy, asthma, osteoarthritis, chronic back pain, obstructive sleep apnoea, non-alcoholic fatty liver disease, and gallbladder diseases (25). Anorectal abscess (ARB) is a suppurative infection resulting from the obstruction of anal glands (35). Clinical manifestations include perianal redness, swelling, pain, and sometimes systemic symptoms such as fever and chills. ARB ranks among the most common diseases in colorectal surgery, with an incidence of 8.6–20 cases per 100,000 individuals, more severe in men than in women (3). In the United States alone, ARB affects approximately 68,000–96,000 patients annually (1).

A retrospective study observed that the risk of ARB was 2.24 times higher in obese patients, and exhibited a J-shaped trend between elevated BMI levels and increased ARB risk (35). Additionally, a study involving 18,877 patients with perianal abscesses from 1997 to 2009 revealed that the ratio between the prevalence of obesity in patients with ARB and the prevalence of the same in the normal population was 1.59 (2). It is worth noting that BMI is also a risk factor of ARB recurrence (22). These studies suggest a link between obesity and ARB.

However, current evidence on the relationship between obesity and ARB is limited and inconclusive, and it has not yet been confirmed whether this association is causal. Due to the susceptibility of randomized controlled trials (RCTs) to confounding factors, which makes it challenging to determine causality between obesity and ARB, it is necessary to employ Mendelian randomization (MR) methods for causal inference. Furthermore, identifying disease risk factors is essential for preventing ARB development. Therefore, it is crucial to determine a causal relationship between obesity and ARB. This study aims to apply MR analysis to determine whether five obesity characteristics—BMI, body fat percentage (BF), whole body fat mass (FM), waist circumference (WC), and hip circumference (HC)—causally influence ARB development.

## Materials and methods

### Data sources and study design

**Figure 1:**
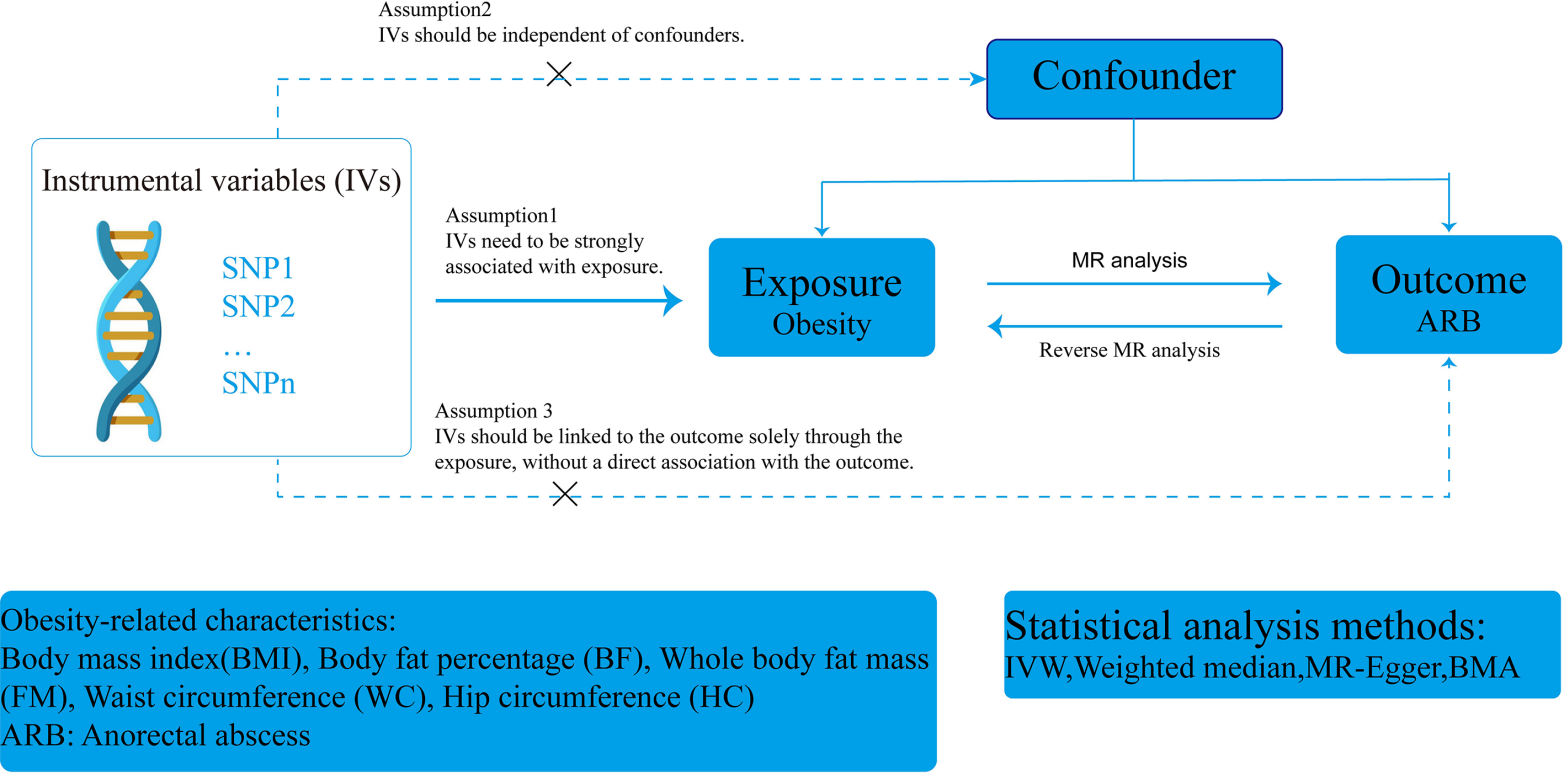
Procedure for an MR analysis.

This study adhered to the STROBE-MR (Strengthening the Reporting of Observational Studies in Epidemiology using Mendelian Randomization) guidelines (Supplementary Table S5) (32). A two-sample MR design was employed using publicly available genome-wide association study (GWAS) data to determine the causal relationship between obesity and ARB. The overall flow chart of this MR study is illustrated in Figure 1. In clinical practice, BMI is commonly used to measure and assess obesity (37). Single nucleotide polymorphisms (SNPs) served as instrumental variables (IVs) to analyze the causal relationship between BMI and ARB using single-factor MR. Subsequently, we assessed the effect of ARB on BMI. Additionally, we evaluated several other obesity-related features for supplemental analysis, including BF, FM, WC, and HC. Finally, due to the high correlation of these obesity-related characteristics with BMI, we reassessed their impact on ARB after adjusting for BMI effects.

The GWAS summary statistics for BMI, obesity-related characteristics, and ARB were sourced from the IEU OpenGWAS database,^1^ which comprises primarily publicly available GWAS data suitable for MR analysis. The BMI GWAS summary involved 461,460 individuals of European descent, while the GWAS summary statistics for ARB were based on 183,710 individuals (1,287 cases and 182,423 controls) within the European population. Detailed information on data sources for obesity-related characteristics is provided in Table 1. All studies received approval from the relevant ethical review committees, and participants provided informed consent.

**Table 1:** Details of the GWASs included in the Mendelian randomization.

Trait	Sample size	Population	GWAS-ID	Consortium	Year
BMI	461,460	European	ukb-b-19953	MRC-IEU	2018
BF	454,633	European	ukb-b-8909	MRC-IEU	2018
FM	454,137	European	ukb-b-19393	MRC-IEU	2018
WC	462,166	European	ukb-b-9405	MRC-IEU	2018
HC	462,117	European	ukb-b-15590	MRC-IEU	2018
ABS	1,287 cases and 182,423 controls	European	finn-b-K11_ABSCANAL	NA	2021

### Selection of instrumental variables

All SNPs used for MR analysis must adhere to three recognized assumptions: (1) the IV must be strongly associated with the exposure, (2) the IV should be independent of confounders, and (3) the IV should affect the outcome solely through the exposure, without a direct association with the outcome (11, 14, 30). Initially, we selected independent SNPs strongly associated with the exposure factors with *p*-values <5 × 10^−8^. For assessing the effect of ARB on BMI, we expanded the *p*-value threshold to less than 5 × 10^−6^ to allow for a sufficient number of IVs. Secondly, to mitigate linkage disequilibrium (LD), SNPs within a window size of 10,000 kb were pruned at a threshold of *r*^2^ < 0.001, ensuring the independence of each IV. We then adjusted the exposure and outcome datasets to exclude SNPs with allelic inconsistencies and SNPs with intermediate allele frequencies (36). Finally, we calculated the *F* statistic to assess the extent of weak instrumental bias, including only IVs with *F* statistics greater than 10 in the MR analysis (29). The equation for the *F*-value used in this study is *F* = Beta^2^/Se^2^, where Beta is the allele effect value, and Se is the estimated standard error of Beta.

### Statistical analysis

Using two-sample MR, we generated estimates of the causal effect of adiposity measures on ARB (OR per SD unit increase). In this MR study, the inverse variance weighting (IVW) method served as the main analytical method for studying causality. The IVW method, an extension of the Wald ratio estimation method based on meta-analysis principles (12), employs the inverse variance of SNPs as weights in meta-analysis to evaluate the combined causal effect. In MR analysis, the IVW is the most effective way to detect causal effects (18). To further demonstrate the stability and directionality of the results, we utilized MR-Egger (7) and the weighted median method (8) for auxiliary assessments of causality. Moreover, due to high correlations among causal genetic markers sharing numerous genetic variants, it was necessary to correct for the influence of “measured polymorphisms.” Thus, Bayesian model averaging (BMA) was applied to further validate the IVW results.

To mitigate the impact of heterogeneity on the causal effect, Cochran’s *Q* test was used to evaluate heterogeneity. If the *p*-value exceeds 0.05, the effect of heterogeneity on the causal effect is considered negligible (10). Considering the influence of unknown confounders on genetic diversity and causal effects, the MR-Egger method was used to test for horizontal pleiotropy (7). Additionally, we employed the Mendelian Randomization Pleiotropy Residual Sum and Outlier (MR-PRESSO) algorithm to detect outliers with significant differences and evaluate adjusted causal effects after removing outliers (34). Finally, a leave-one-out sensitivity test was used to assess the validity and stability of the MR results.

All analyses were performed using R software (version 4.3.1) and the “TwoSampleMR” and “MRPRESSO” packages for MR analysis. *p* < 0.05 was considered statistically significant. As this study constitutes a secondary analysis of publicly available data, ethical approval was not required.

## Results

### Causal effects of BMI on ARB

**Figure 2:**
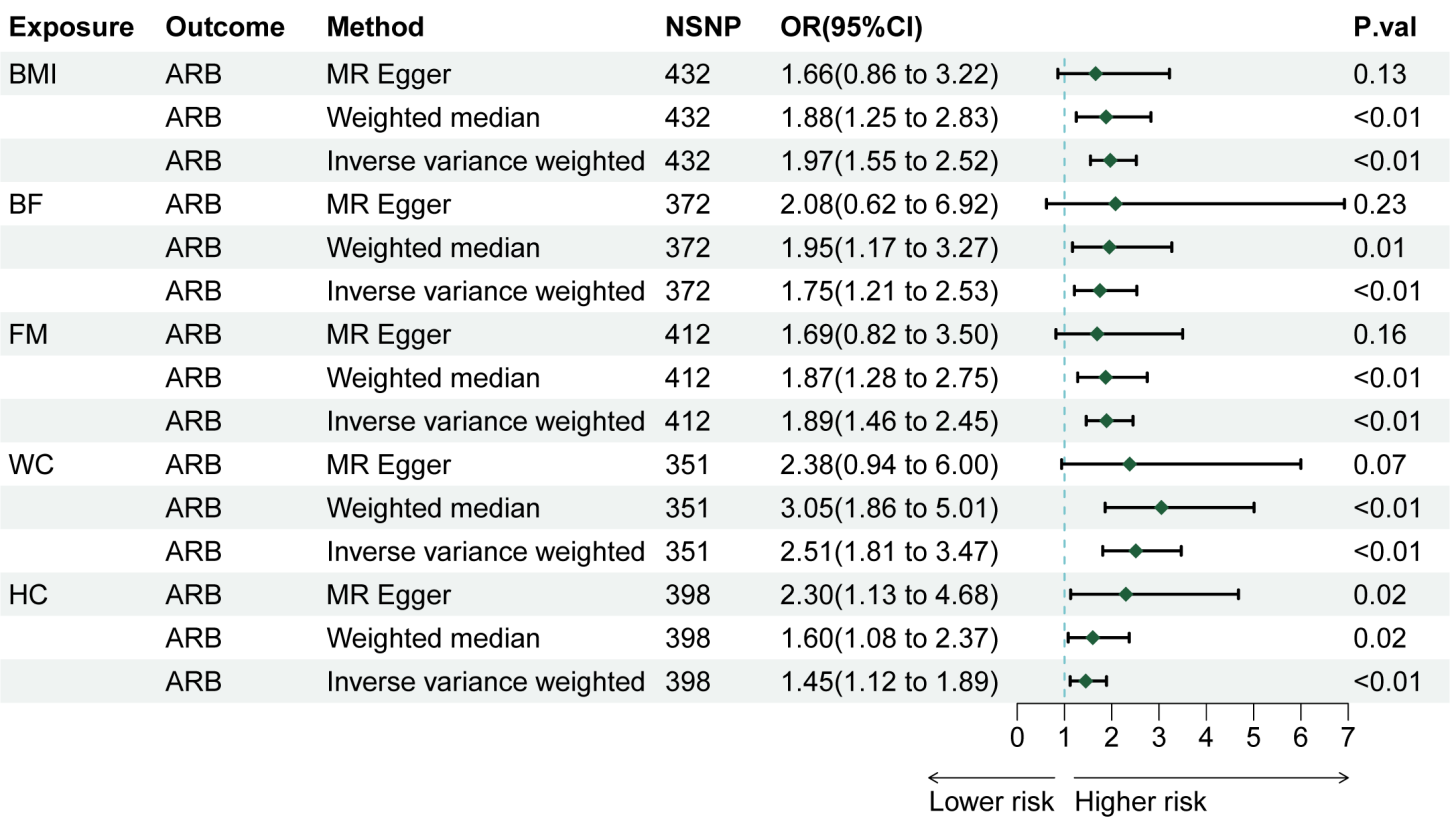
The risk association between obesity and anorectal abscess in a forest plot.

**Figure 3:**
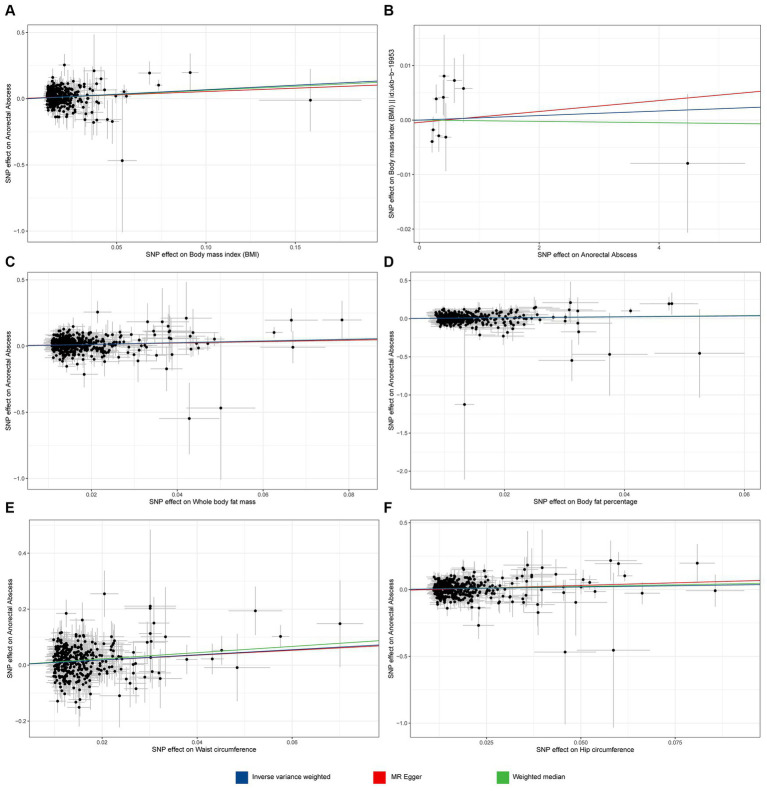
Scatter plot of MR analyses from obesity to anorectal abscess. **(A)** Body mass index (BMI) to anorectal abscess (ARB). **(B)** ARB to BMI. **(C)** Whole body fat mass to ARB. **(D)** Body fat percentage to ARB. **(E)** Waist circumference to ARB. **(F)** Hip circumference to ARB.

Through rigorous screening of SNPs, we identified 432 SNPs strongly associated with BMI. The *F* statistic for each SNP was greater than 10, as detailed in Supplementary Table S1. For each 1-SD kg/m^2^ increase in genetically determined BMI, the IVW indicated an increased risk of ARB [odds ratio (OR) = 1.974, 95% confidence interval (CI): 1.548–2.519, *p* = 4.34 × 10^−8^ Figure 2]. The weighted median method (OR = 1.879, 95% CI 1.248–2.829, *p* = 0.002) and BMA (OR = 1.88, 95% CI 1.477–2.392, *p* = 2.85 × 10^−7^) also showed consistent results (as detailed in Supplementary Tables S2, S4). Sensitivity analysis tests, including Cochran’s *Q* test and MR-Egger test, detected neither heterogeneity nor horizontal pleiotropy (Supplementary Table S3). Moreover, no outliers were detected in the MR-PRESSO test. The results of the leave-one-out sensitivity test indicated that no individual SNP significantly influenced the causal relationship between BMI and ARB (Supplementary Figure S1). Overall, the MR study results affirm a significant causal link between BMI and ARB, which is positively correlated. The estimated effect sizes for the genetic predictions are presented as scatter plots (Figure 3).

Further analysis assessed whether ARB causally affects BMI. Results from IVW, MR-Egger, and the weighted median method consistently indicated that ARB had no effect on BMI (Supplementary Table S2).

### Causal effects of obesity-related characteristics on ARB

We also explored the causal relationship of obesity-related characteristics (WC, HC, BF, and FM) with ARB. MR analysis showed that WC (IVW, OR = 2.509, 95% CI 1.814–3.471, *p* = 2.73 × 10^−8^), HC (IVW, OR = 1.454, 95% CI 1.121–1.887, *p* = 0.004), BF (IVW, OR = 1.747, 95% CI 1.206–2.530, *p* = 0.003), and FM (IVW, OR = 1.891, 95% CI 1.462–2.447, *p* = 1.23 × 10^−6^) are causally related to an increased risk of ARB (Figure 2). We used BMA to further validate the results of the two-sample MR, as shown in Supplementary Table S4, and the BMA results were consistent with the two-sample MR, which indicated that our two-sample Mendelian randomization results were reliable. Sensitivity analyses, including Cochran’s Q test, did not reveal any evidence of heterogeneity (Supplementary Table S3). Additionally, no horizontal pleiotropy was detected by the MR-Egger method. Although outliers were detected in MR-PRESSO tests for HC and BF, these factors remained significantly associated with ARB after outlier removal (Supplementary Table S2). Given the high correlation of these characteristics with BMI, multivariate MR analyses adjusting for BMI were performed. Post-adjustment, none of the obesity-related characteristics demonstrated a significant causal relationship with ARB (Supplementary Table S2).

## Discussion

Our MR analysis established a causal relationship between obesity and an increased risk of ARB, highlighting the significant role obesity plays in the development of ARB. In our study, we considered not only total obesity as measured by BMI but also specific obesity measures. WC and HC serve as indicators of central obesity, while BF and FM represent total body fat (19). Including these indicators could provide deeper insights into the relationship between obesity and ARB.

Our findings align with those of previous observational studies that have examined the association between obesity and ARB. Studies in European populations have noted a higher prevalence of ARB among obese individuals compared to the general population, along with a notable increase in ARB recurrence rates among obese patients over time (2). Additionally, a retrospective study conducted in Asia indicated that the risk of perianorectal abscesses progressively increased with rising BMI (35). These observational studies demonstrate a clear association between obesity and ARB. However, such studies are prone to biases, including confounding and reverse causation, due to limitations inherent in their design and conventional statistical methods. Thus, we utilized a two-sample MR analysis to verify if the observed associations between obesity and ARB were indeed causal.

The association between obesity and ARB may be linked to decreased immunity. Obesity can cause a rapid increase in adipose tissue (AT). The rate of angiogenesis may not keep up with AT expansion, leading to local hypoxia and fibrosis in AT (13, 20, 33). These changes promote the release of inflammatory factors and enhance the local inflammatory response, ultimately leading to fat cell dysfunction, metabolic alterations, and reduced immunity (9, 20, 24).

The oxygen deficit in AT prompts fat cells to undergo anaerobic glycolysis, which promotes the production and release of lactic acid, further promoting the inflammatory pathway in macrophages and leading to chronic inflammation (28, 31). Recent studies suggest that prolonged chronic inflammation can significantly impair the proliferative capacity of hematopoietic stem cells—the progenitors of immune cells—resulting in cumulative and irreversible functional damage and long-term suppression of hematopoietic cells, further leading to immune cell failure and a decline in immune function (6). It has been observed that macrophages in obese individuals shift from an anti-inflammatory M2 phenotype to a pro-inflammatory M1 phenotype (4), and these macrophages exhibit reduced phagocytic ability and reduced capacity to clear bacteria (15). For instance, leptin-deficient mice, characterized by increased food intake and body weight (38), have macrophages that are less capable of clearing pathogens such as *Escherichia coli*, *Candida albicans*, or *Klebsiella pneumoniae* (5, 23). Notably, *E. coli* is the primary pathogen isolated from the pus of ARB, while *Klebsiella pneumoniae* is frequently found in diabetic patients (21). Obesity also hampers T lymphocyte receptor diversity, disrupts normal antigen presentation processes, decreases T lymphocyte response efficiency, and increases the susceptibility of obese individuals to infections (15). Obesity contributes to the downregulation of neutrophil function, which impairs bacterial clearance (15, 23). Long-term systemic inflammation in obese patients can weaken immunity and reduce the capability of immune cells to eliminate bacteria, thus elevating the risk of bacterial infections and potentially leading to ARB. Additionally, obese patients often exhibit abnormal lipid and glucose metabolism; high blood sugar and lipid levels can predispose the anal sinuses and glands to infections, which may progress to abscesses as pus moves from the anal glands to the intersphincteric space (35).

Furthermore, our study examined the link between various obesity-related characteristics and the risk of ARB. Univariate MR analysis indicated that WC, HC, BF, and FM were positively associated with the risk of ARB. However, these associations seemed to be mediated through the effects of BMI, as no significant causal links were observed after adjusting for BMI. While our findings are derived solely from MR methods, further clinical studies are necessary to confirm these relationships. Based on our results, we recommend using BMI for ARB risk assessment in clinical practice.

To our knowledge, this is the first MR study to assess the causal relationship between obesity and ARB. However, there are limitations to consider. Firstly, as our study used GWAS data from European populations, caution should be exercised when generalizing these findings to other ethnic groups. Secondly, our analysis did not stratify by age and sex due to database limitations; future studies should investigate how obesity affects ARB risk across different demographics. Lastly, the impact of obesity on the severity of ARB was not addressed and should be explored in future clinical observational studies.

## Conclusion

Our MR study confirms a positive causal relationship between genetically predicted increased BMI and the risk of ARB, improving our understanding of how obesity can lead to ARB and informing preventive and therapeutic strategies.

## Data availability statement

The original contributions presented in the study are included in the article/Supplementary material, further inquiries can be directed to the corresponding author.

## Author contributions

XZ: Conceptualization, Data curation, Methodology, Software, Writing – original draft. HW: Data curation, Software, Writing – original draft. YD: Data curation, Software, Validation, Visualization, Writing – original draft. ZD: Visualization, Writing – original draft. WB: Validation, Writing – review & editing. HF: Supervision, Validation, Writing – review & editing.

## Footnotes

^1^ https://gwas.mrcieu.ac.uk/
