# Supplementary material for: Causal relationship between obesity and anorectal abscess: a Mendelian randomization study
Source: Front Med (Lausanne). 2024 Jun 21;11:1437849. doi: 10.3389/fmed.2024.1437849 (PMC11225408; doi:10.3389/fmed.2024.1437849)

Supplementary Figure

**Supplementary Figure S1.** Leave-one-out plot and Funnel plot of MR analyses from Body mass index to Anorectal abscess.


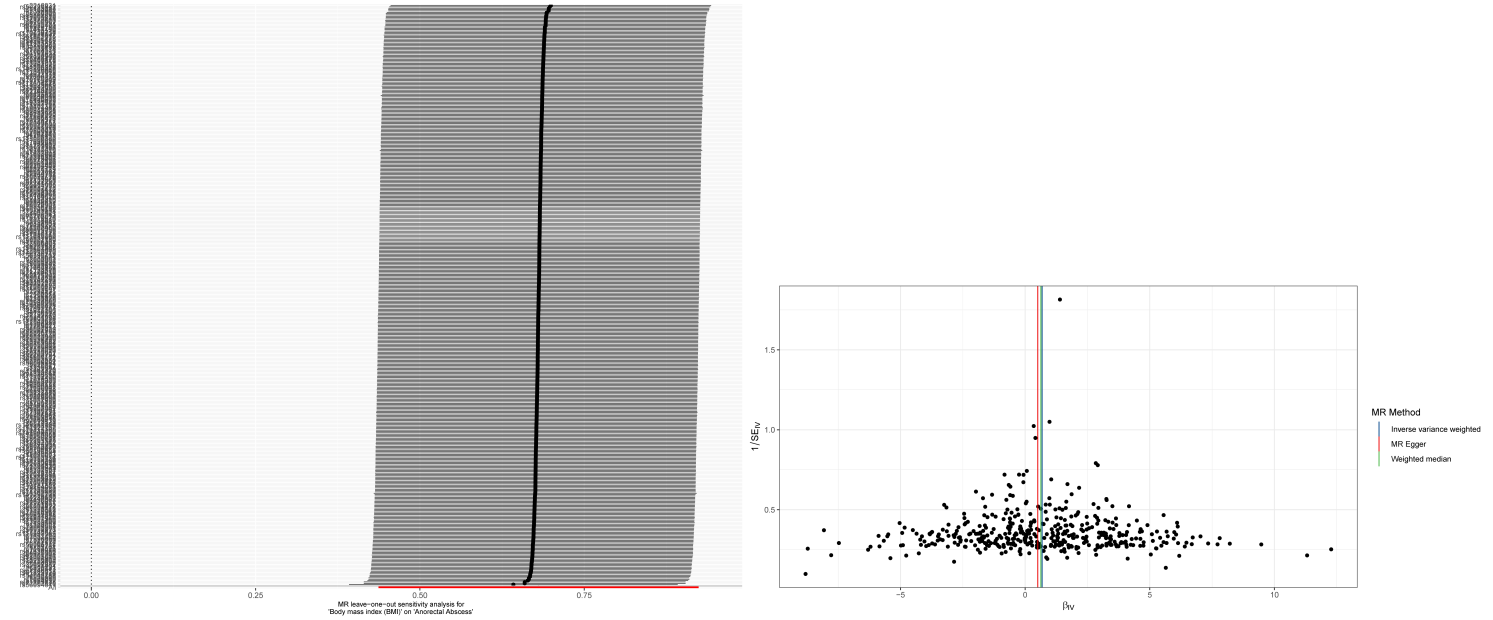


**Supplementary Figure S2.** Leave-one-out plot and Funnel plot of MR analyses from Anorectal abscess to Body mass index.


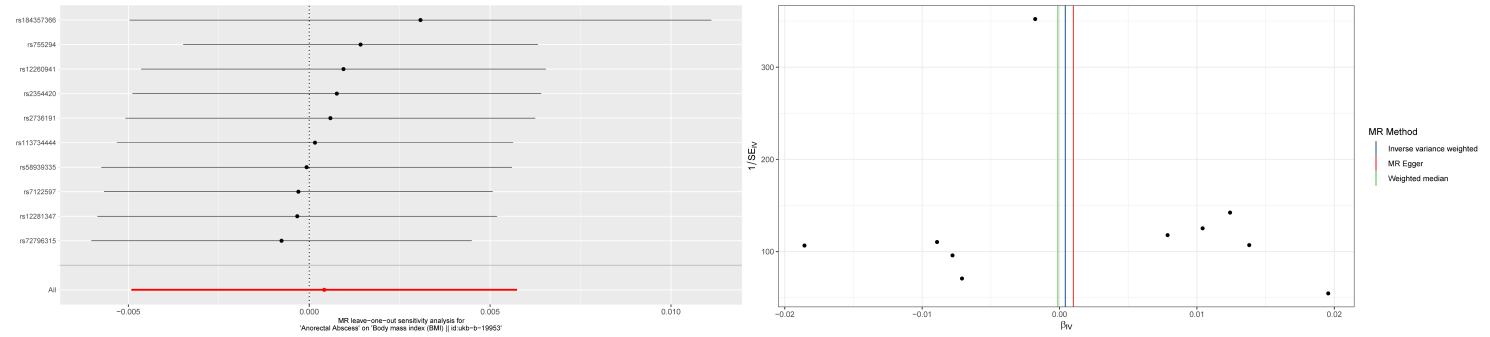


**Supplementary Figure S3.** Leave-one-out plot and Funnel plot of MR analyses from Body fat percentage to Anorectal abscess.


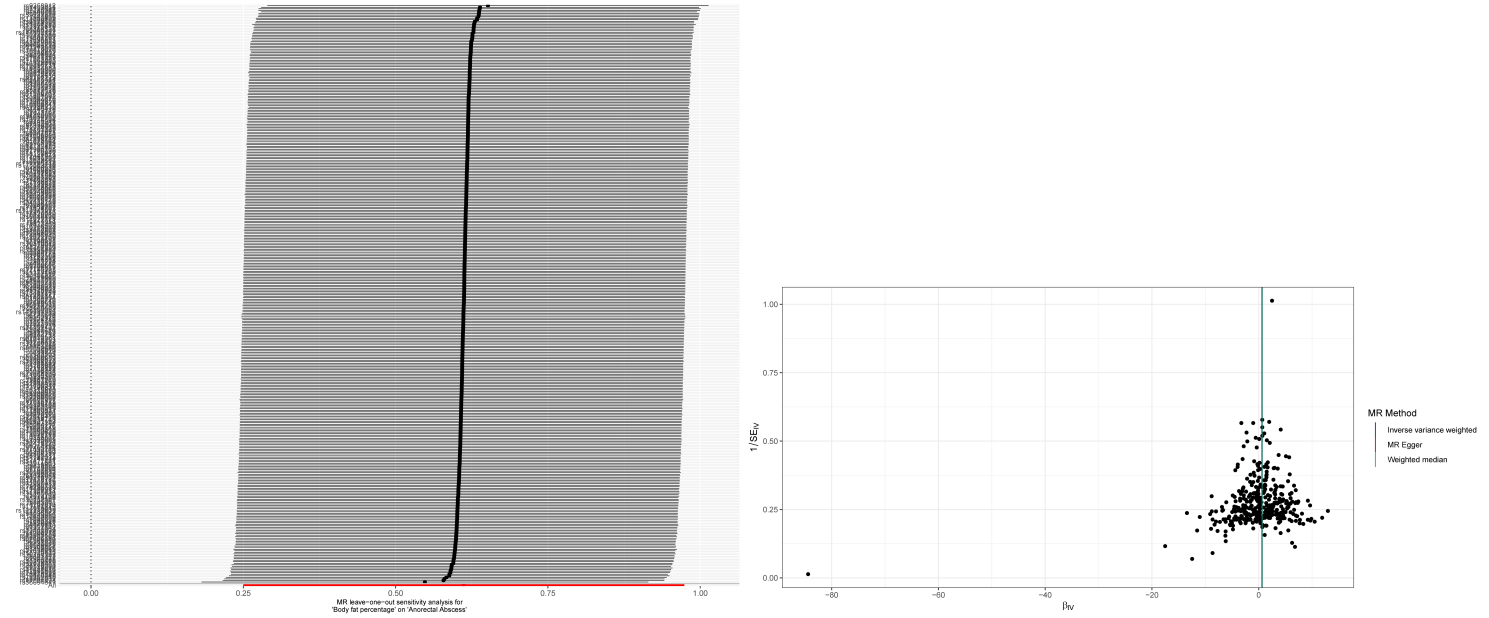


**Supplementary Figure S4.** Leave-one-out plot and Funnel plot of MR analyses from Whole body fat mass to Anorectal abscess.


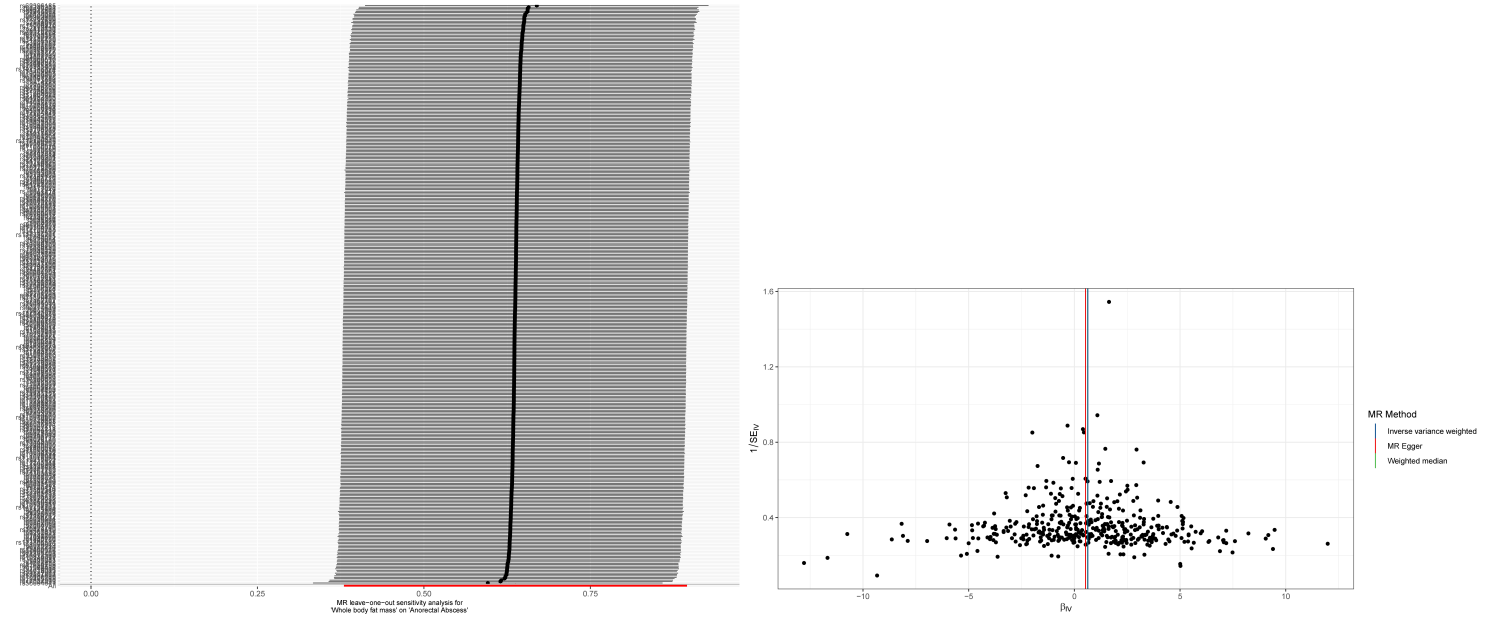


**Supplementary Figure S5.** Leave-one-out plot and Funnel plot of MR analyses from Waist circumference to Anorectal abscess.


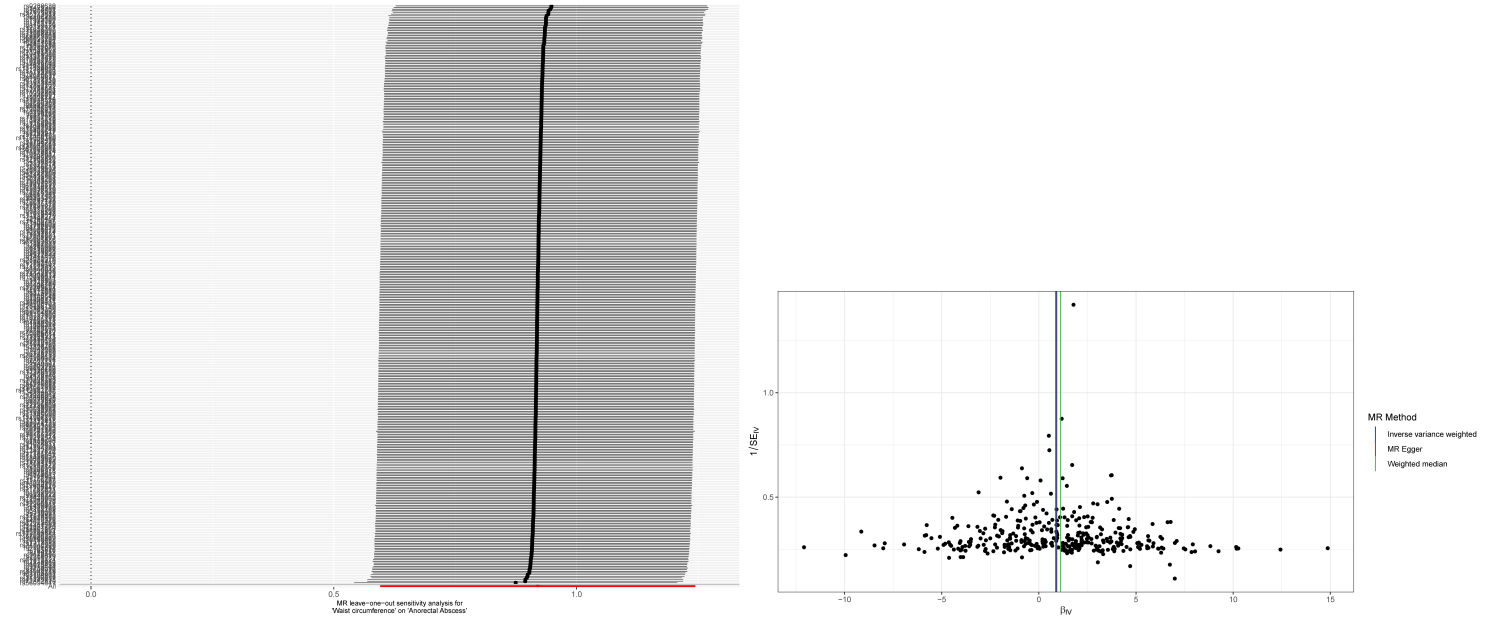


**Supplementary Figure S6.** Leave-one-out plot and Funnel plot of MR analyses from Hip circumference to Anorectal abscess.


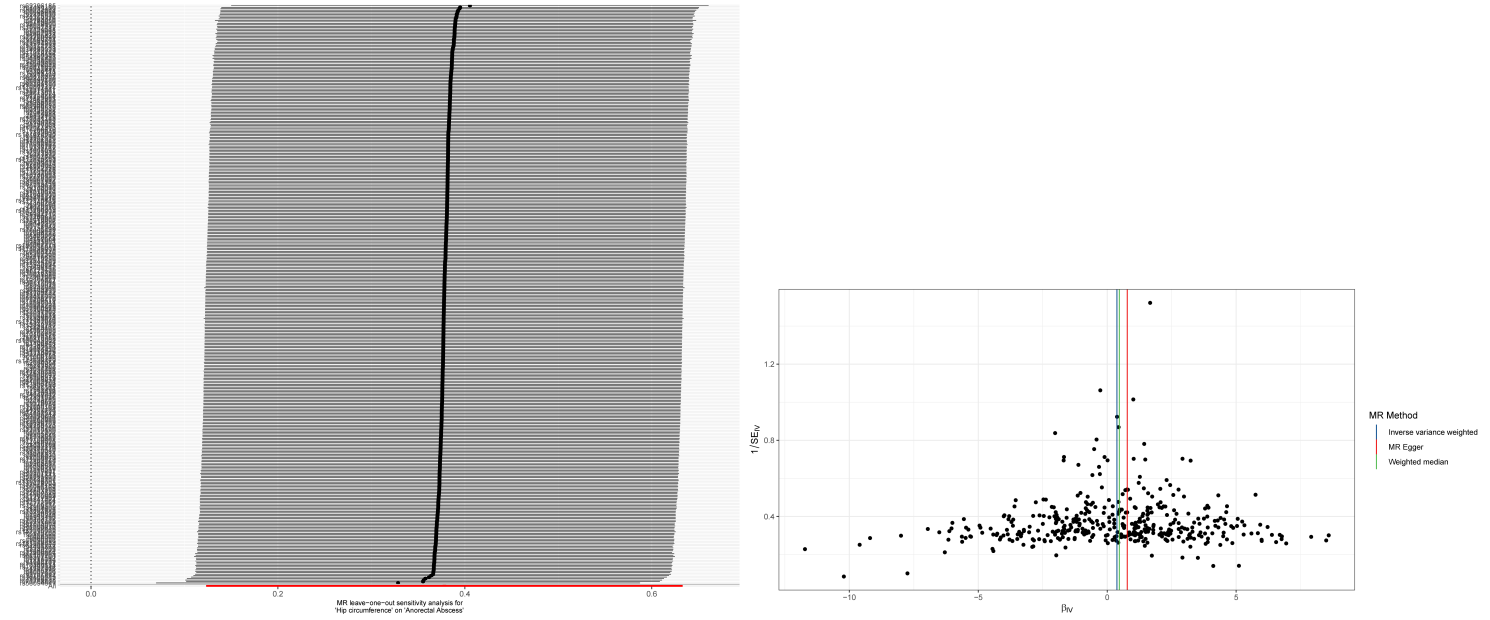

Supplement: Supplementary file 6 [file Table_6.docx]
